# *Sarcandra glabra* Extract Reduces the Susceptibility and Severity of Influenza in Restraint-Stressed Mice

**DOI:** 10.1155/2012/236539

**Published:** 2012-11-01

**Authors:** Hui-Juan Cao, Rui-Rong Tan, Rong-Rong He, Lu-Ping Tang, Xin-Luan Wang, Nan Yao, Wen-Jun Duan, Yuan-Ao Hu, Xin-Sheng Yao, Hiroshi Kurihara

**Affiliations:** ^1^Department of Medicine, Institute of Traditional Chinese Medicine and Natural Products, Jinan University, Guangzhou 510632, China; ^2^Institute of Biomedical and Health Engineering, Translational Medicine Research and Development Center, Shenzhen Institutes of Advanced Technology, Chinese Academy of Sciences, Shenzhen 518055, China; ^3^State Key Laboratory of Drug Research, Shanghai Institute of Materia Medica, Chinese Academy of Sciences, Shanghai 201203, China; ^4^Pharmacology Laboratory of Traditional Chinese Medicine, Guangdong Provincial Institute of Traditional Chinese Medicine, Guangzhou 510095, China

## Abstract

*Sarcandra glabra*, as a type of “antipyretic-detoxicate drugs”, has always been widely used in traditional Chinese medicine (TCM). The *Sarcandra glabra* extract (SGE) is applied frequently as anti-inflammatory and anti-infectious drug in folk medicine. However, relative experiment data supporting this effective clinical consequence was limited. In order to mimic the physiological conditions of the susceptible population, we employed restraint stress mouse model to investigate the effect of SGE against influenza. Mice were infected with influenza virus three days after restraint, while SGE was orally administrated for 10 consecutive days. Body weight, morbidity, and mortality were recorded daily. Histopathologic changes, susceptibility genes expressions and inflammatory markers in lungs were determined. Our results showed that restraint stress significantly increased susceptibility and severity of influenza virus. However, oral administration of SGE could reduce morbidity, mortality and significantly prolonged survival time. The results further showed that SGE had a crucial effect on improving susceptibility markers levels to recover the balance of host defense system and inhibiting inflammatory cytokines levels through down-regulation of NF-*κ*B protein expression to ameliorate the lung injury. These data showed that SGE reduced the susceptibility and severity of influenza.

## 1. Introduction

Influenza is an acute respiratory disease caused by influenza virus that seriously threatens human lives. Most people will completely recover from influenza without other special treatment apart from antipyretic medication and rest. However, some patients will develop other symptoms and complications, such as croup, arthralgia, chest pain, lymphadenopathy, and even pneumonia. Pneumonia can develop rapidly and may result in respiratory failure and death [1, 2]. Despite many chemical antiviral drugs have been used clinically, the emergence of influenza virus drug resistant and subsequent viral pneumonia is still the leading causes of morbidity and mortality in influenza patients [3, 4]. Therefore, more attention has been paid to explore natural and active substances on anti-influenza.

*Sarcandra glabra *(Thunb.) Nakai (Chloranthaceae) is widely used in TCM as antipyretic, detoxicate, antitumor, anti-inflammatory, and anti-infectious drug [5]. *Sarcandra glabra* extract (SGE) contains high level of rosmarinic acid, isofraxidin, fumaric acid, terpenoid saponins, and others. It has been showing potential effect in the therapy of influenza and its symptoms in clinical practice [5]. However, there was only little data about the anti-influenza effect of SGE since the use of nonsuitable animal model and positive result was difficult to obtain through in vitro antiviral experiments. This paradox can be explained if host factors are important in the pathogenesis and outcome of influenza infection. It is well known that influenza virus killed millions of people in the 20th century [6]. Although influenza virus infected a wide range of people, the ones most likely to need hospitalization are the newborns, elderly, the sick, and individuals presenting fatigue or stress, who have weakened immunity. It has been reported that over 90% of deaths had occurred in these susceptible population [7, 8]. Stress has been shown to increase susceptibility to viral or bacterial pathogens, influence the severity of infectious disease, and diminish the strength of host immune defense [9, 10]. The reason for using restraint stressed mice model is because restraint is a commonly used stressor. Our previous studies had indicated that restraint stress might enhance the risk of developing infectious disease and also prolong infectious illness episodes [10], which is more conducive to simulate the clinical features of susceptible population. We had also successfully used restraint stress mice model to evaluate anti-influenza effects of natural products [10]. Therefore, we employed restraint stress mice model to investigate the effects of SGE on influenza and its subsequent pneumonia. 

## 2. Materials and Methods

### 2.1. Preparation of SGE

**Figure 1:**
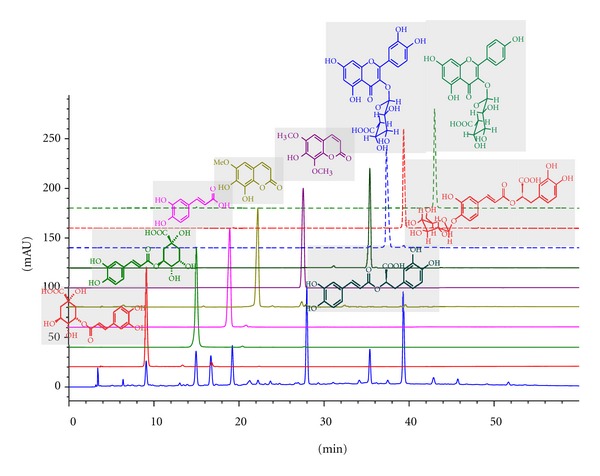
Chemical fingerprint of SGE. Each peak of SGE in the HPLC fingerprint was identified by comparison of the retention times and UV spectra of chemically defined standard compounds. The standard compounds were isolated from SGE previously. Several batches of SGE were analyzed, and similar profiles were observed. HPLC condition was as follows: agilent series 1100 HPLC; column, Welch material XB-C18, 4.6 × 250 mm, particle size 5 mm; mobile phase A, H_2_O with 0.2% HAc; mobile phase B, MeOH with 0.2% HAc; elution program: 20% B in 5 min, linear gradient from 20% B to 62% B in 55 min, 100% B in 12 min; flow rate at 0.80 mL/min; detection wavelength at 330 nm; injection volume in 10 *μ*L and oven temperature at 35°C.

*Sarcandra glabra* extract (no. 090303) was provided by Guangzhou Jingxiutang Pharmaceutical Co. Ltd. (Guangzhou, China). SGE was qualitatively analyzed employing HPLC as previously reported [11]. As shown in Figure 1, the main chemical ingredients of SGE were 5-caffeoylquinic acid, 3-caffeoylquinic acid, 4-caffeoylquinic acid, caffeic acid, isofraxidin, 4′-O-*β*-D-glycopyranosyl rosmarinic acid, and rosmarinic acid. Rosmarinic acid and isofraxidin were used as marker for the quality control of SGE. Each gram of SGE contained 2.4 mg of rosmarinic acid and 3.5 mg of isofraxidin [12]. 

### 2.2. Animals and Virus

Male specific pathogen free Kunming mice (13–15 g) were purchased from Guangdong Medical Laboratory Animal Center (Guangzhou, China). All mice were kept in a pathogen-free animal room under controlled temperature at 23 ± 1°C. A 12 h light-dark cycle was maintained, with lights on from the time 06:00 to 18:00. The mice were provided with standard laboratory diet and water. All studies were conducted in accordance with the guidelines set by the National Institutes of Health and the U.S. Department of Agriculture (publication number 85-23, revised 1996). The influenza A/FM/1/47 (H1N1) virus was provided by the College of Veterinary Medicine of South China Agricultural University (Guangzhou, China). The virus strain was propagated in specific pathogen-free fertilized eggs and adapted for lethality in mice after three passages in the animal. Virus containing allantoic fluid was harvested and stored in aliquots at −80°C. The LD50 was determined in mice after serial dilution of the stock. Two times value of LD50 were used for viral challenge in all of the experiments. Infection was established by intranasal inoculation in mice anesthetized by ethyl ether. The influenza-related pathogenic operation was performed in the Animal Biosafety Level 3 Laboratory in South China Agriculture University.

### 2.3. Experimental Design

 The experimental mice were randomly divided into six groups: normal control, virus control (virus only), model control (restraint + virus), positive control (restraint + virus + 50 mg/kg ribavirin), and two SGE groups (restraint + virus + 250 or 500 mg/kg SGE). Ribavirin and SGE were administered to mice by oral gavage for 10 consecutive days, while the rest of the groups received water only. On the 2nd day of administration, mice were physically restrained in a 50 mL polypropylene centrifuge tube with holes for 18 h. After recovery for three day, the animals were anesthetized by inhalation of ether vapor and then an approximate 2 × LD50 amount of virus (30 *μ*L) was instilled into the nares. 

Experiment was conducted in triplicates of 9–11 mice for each group to observe daily changes in body weight, survival, and several typical symptoms of illness, including ruffled fur, redness around the eyes, nose or mouth, hunched back, altered respiration and unresponsiveness, for 21 days or until death. Another experiment was conducted in duplicates of 8 mice for each group. On the 5th day after virus infection, mice were weighed and anesthetized by ethyl ether. Blood was obtained by cardiac puncture and lungs were removed and weighed. 

### 2.4. Histopathologic Analysis

To monitor the histological changes in the lungs of influenza virus-infected animal, all mice were sacrificed for determination of lung index. Simultaneously, lung tissues were immediately fixed in 4% buffered formalin and embedded in paraffin wax. Lung sections (4–6 *μ*m) were sliced by slicer (Leica) and mounted on microscopic slides. Histopathologic changes were examined by H&E staining under a light microscope (Olympus) as described.

### 2.5. Measurement of NO Level in Serum

 Blood samples were collected and centrifuged at 500 ×g for 15 min to obtain serum. Serum NO levels were determined by Griess test [13, 14]. 

### 2.6. Reverse Transcription Polymerase Chain Reaction (RT-PCR)

Total RNA in lysed lung tissues was extracted as previously described [10] and reversely-transcribed to cDNA by applying mouse moloney leukemia virus reverse transcriptase (Invitrogen). Viral nucleoprotein (NP) gene, SP-A, SP-D, ICAM-1, IFITM 3, IL-1*β*, TNF-*α* and iNOs mRNA levels in lung tissues and internal control 18S gene were measured by the Veriti PCR System (Applied Biosystems), using primers listed in Table 1. The PCR products were fractionated on a 1% agarose gel and visualized by ethidium bromide staining. The band intensity of ethidium bromide fluorescence was measured using an image analysis system (Bio-Rad, Hercules, CA), then quantified with Quantity One analysis software (Bio-Rad, Hercules, CA), and expressed as the ratio to 18S.

### 2.7. Western Blotting

 The cytosolic and nuclear proteins were harvested from lung sample using extraction kit according to the protocol of the manufacturer (KeyGEN Biotech, China). Samples were run on a 10% SDS/PAGE gel and electroblotted onto nitrocellulose membranes. Immunoblotting was assayed using anti-NF-*κ*B p65 (1 : 2000) antibodies (Cell Signaling Technology, Inc.). The immunodetection was done using an enhanced chemiluminescence detection kit (MultiSciences Biotech Co., Ltd., China). The bands density was quantified using Quantity One analysis software (Bio-Rad, Hercules, CA) via calculating the average optical density in each field.

### 2.8. Statistical Analysis

The data were presented as mean ± standard error (SE). Statistical comparisons of data were carried out using the ANOVA of the SPSS 19.0 system. A value of *P* < 0.05 was considered significant. Differences in morbidity (time to sickness) and mortality (time to death) between groups across the 21-day postinfection period were determined using the Lifetest Survival Analysis program in SigmaStat (*P* < 0.05), which analyzed mean and median time to sickness and death.

## 3. Results

### 3.1. Effects of SGE on Influenza Caused Morbidity and Mortality in Restraint-Stressed Mice

**Figure 2:**
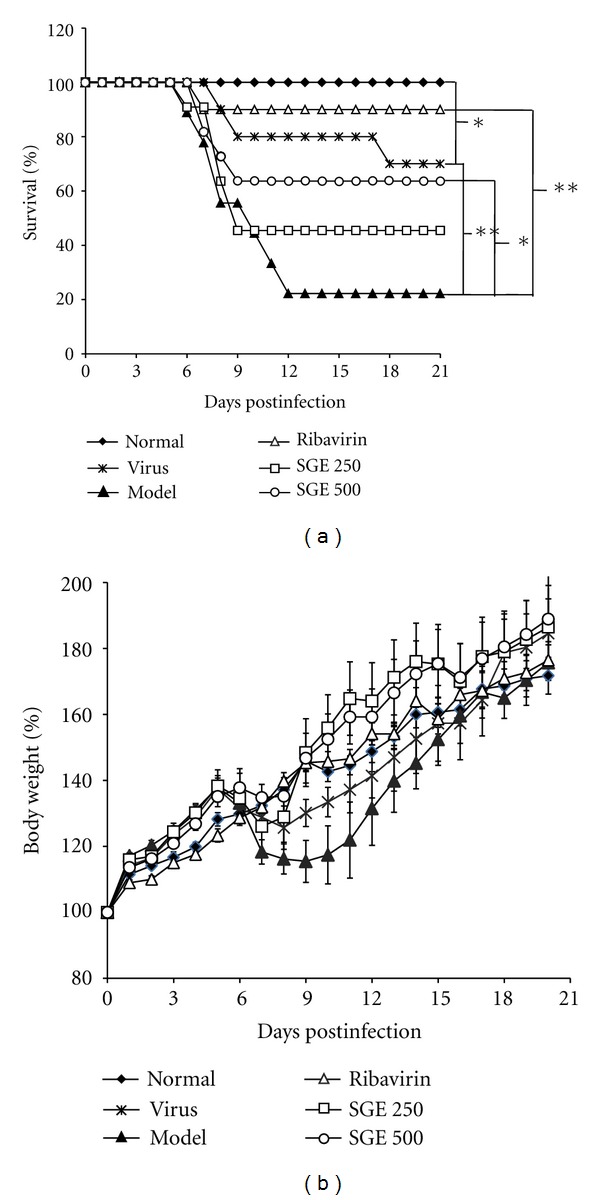
Effect of SGE on survival rates (a) and body weight changes (b) of restraint-stressed mice after infection. Three days before H1N1 virus infection, Kunming mice were fixed in a restraint cage for 18 h. Time course of mortality and survival days of each mouse were recorded until the 21st day after viral infection. Data were obtained from 9–11 animals in each group. **P < 0.01, *P < 0.05.

 The effects of SGE against H1N1 influenza were evaluated in restraint stressed mice plus viral infection. After intranasal inoculation of influenza virus, mice were monitored daily for survival and weight changes. A significantly lower survival rate was observed in model control group as compared to virus control (*P* < 0.01, Figure 2(a)). Only 22% of mice in the model control group survived and the mean day to death (MDD) was decreased from 18.2 ± 1.9 to 11.5 ± 1.9 day when compared to virus control group (*P* < 0.01). Ribavirin (50 mg/kg) significantly improved the survival rate to 90% and MDD to 19.6 ± 1.4 day (*P* < 0.01). SGE also demonstrated inhibitory effects against virus-induced death, with 45% and 64% of mice surviving in the 250 and 500 mg/kg/day SGE treatment groups, respectively. The MDD of 500 mg/kg/day SGE was also prolonged to 16.1 ± 2.2 day (*P* < 0.05). Thereafter, the body weight of virus infected mice began to decrease at day 5 and dropped to a minimum at day 7 (Figure 2(b)). Survived virus infected mice started to gain weight on day 8, but model control mice kept losing weight until day 10. SGE treatment at 250 and 500 mg/kg/day could significantly improve health status of virus infected mice. Body weight changes were rather stable when compared to other groups, with slight weight loss at day 6. Furthermore, behavioral changes, such as a tendency to huddle, ruffed fur, altered respiration, and reduced food intake were observed in infected mice on the 6th day after virus infection. Morbidity was presented as percentage of morbid mice to total number of mice. As shown in Table 2, group differences in morbidity were evident over the 21-day post-infection period. Mice fixed in a restraint cage for 18 h resulted in an increase in morbidity compared with virus control (*P* < 0.05). Model control experienced a 100% incidence in morbidity, while only 80% for virus control group. Mean time to sickness was 7.2 ± 0.2 day for model control and 10.5 ± 1.8 day for virus control. SGE treatment at 250 and 500 mg/kg/day significantly alleviated influenza symptoms and offset in morbidity associated with restraint stress (*P* < 0.05). Incidence of morbidity was both 82% in 250 and 500 mg/kg/day SGE groups, while mean time to sickness was 10.0 ± 1.6 and 9.7 ± 1.7 day, respectively. These results indicated that SGE improved survival rates and prolonged survival time of virus infected stressed mice in a dose-dependent manner.

### 3.2. Effects of SGE on Influenza-Caused Pneumonia in Restraint-Stressed Mice. 

**Figure 3:**
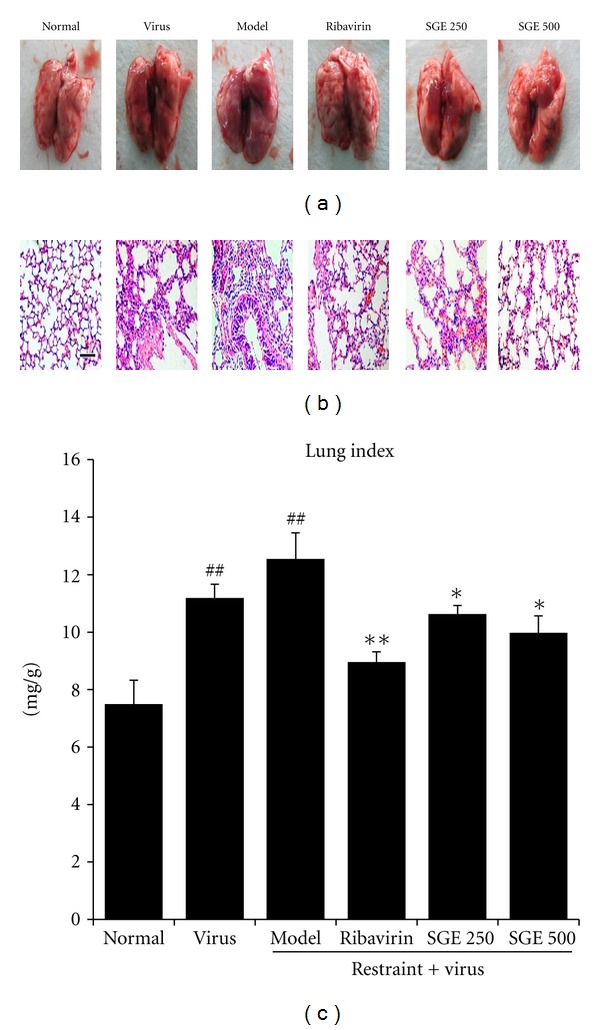
Mice showed few visible signs of external damage in lung lobes on day 5 postinfection. (a) Intact lungs. (b) Histopathologic changes in lung tissues collected at the 5th days postinfection. Representative histologic sections of lung tissues from experimental mice were stained by H&E, bar = 100 *μ*m. (c) Lung index was calculated according to the following formula: lung index = lung weight (mg)/body weight (g). The results represented the mean ± SE of values obtained from 8 Kunming mice in each group. The significance of differences was from the normal control group at ^##^ *P* < 0.01 and from the model group at **P < 0.01, *P < 0.05.

As shown in Figure 3(a), on day 5 after infection, lungs were characterized by extensive edema, red blood cell extravasation, and inflammation. Part of the bronchial mucosa had shedding or erosion. Moreover, large lesions on all lung lobes were found in model group. The lung index was used to evaluate edema level caused by the influenza virus. As shown in Figure 3(c), the lung index baseline of normal control was 7.5 ± 0.8 mg/g. It was increased to 11.2 ± 1.5 mg/g (*P* < 0.01) for virus control and was further increased to 12.5 ± 1.9 mg/g (*P* < 0.01) in model control. In comparison to model control, ribavirin significantly recovered the lung index to 9.0 ± 0.3 mg/g (*P* < 0.01). Moreover, SGE (250 and 500 mg/g/day) also recovered lung index to 10.6 ± 3.3 and 9.9 ± 0.6 mg/g (*P* < 0.05). The effects of SGE on the pathogenesis of influenza virus-induced pneumonia in stressed mice were observed by H&E staining (Figure 3(b)). In lungs with pneumonia, there was more inflammatory cells infiltration. Alveoli were filled with hemorrhage exudates and alveolar wall was thickened. Moreover, the lung tissues of mice in model group around the lesions showed significant compensatory emphysema and bronchial epithelial hyperplasia. However, the lung pathogeneses in SGE and ribavirin groups were significantly reduced compared with model group.

### 3.3. Effects of SGE on the Susceptibility Genes to Influenza Infection in Restraint-Stressed Mice

**Figure 4:**
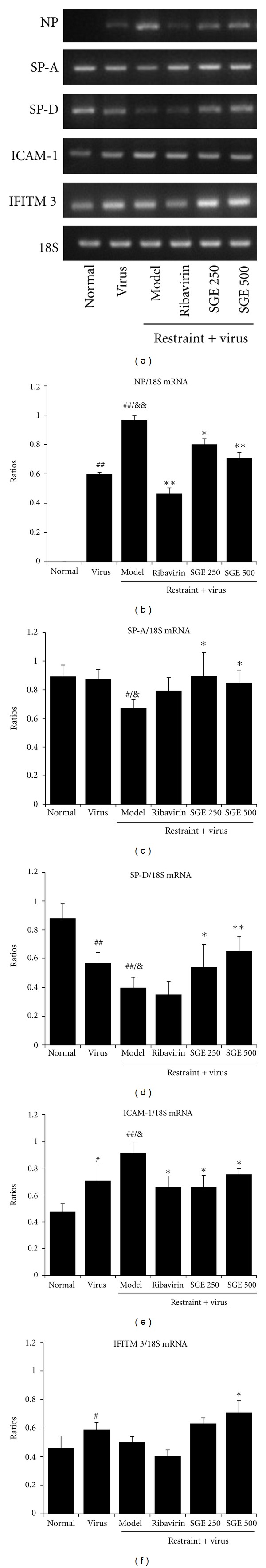
Effect of SGE on susceptibility gene expressions in restraint-stressed mice. Viral NP, SP-A, SP-D, ICAM-1 and IFITM 3 mRNA expressions in lung tissues from 8 mice in each group were determined by RT-PCR and normalized by 18S. The significance of differences was from the normal control group at ^##^ *P* < 0.01, ^#^ *P* < 0.05, from virus control group at ^&&^ *P* < 0.01, ^&^ *P* < 0.05, and from the model group at **P < 0.01, *P < 0.05.

 RT-PCR was used to explore if SGE would reduce susceptibility to influenza infection in restraint-stressed mice. NP mRNA level was examined as an indicator of viral replication and clearance degree in the lungs. As shown in Figure 4, NP mRNA expression in the lungs of model mice was nearly two fold higher than that in virus control group (*P* < 0.01). However, SGE treatment (250, 500 mg/kg/day) obviously decreased NP mRNA level (*P* < 0.05, *P* < 0.01). In addition, SP-A, SP-D, ICAM-1, and IFITM3, which were known to be crucial in host defense and affect virus replication and clearance capability in lung, were also examined. The mRNA expression of ICAM-1 was increased, while SP-A, SP-D, and IFITM3 were significantly decreased in restraint stress mice when compared to virus control. Administration of SGE (250, 500 mg/kg/day) obviously decreased ICAM-1 mRNA level and increased SP-A, SP-D and IFITM 3 mRNA expression.

### 3.4. Effects of SGE on Influenza-Caused Inflammatory Cytokine Production in Restraint-Stressed Mice

**Figure 5:**
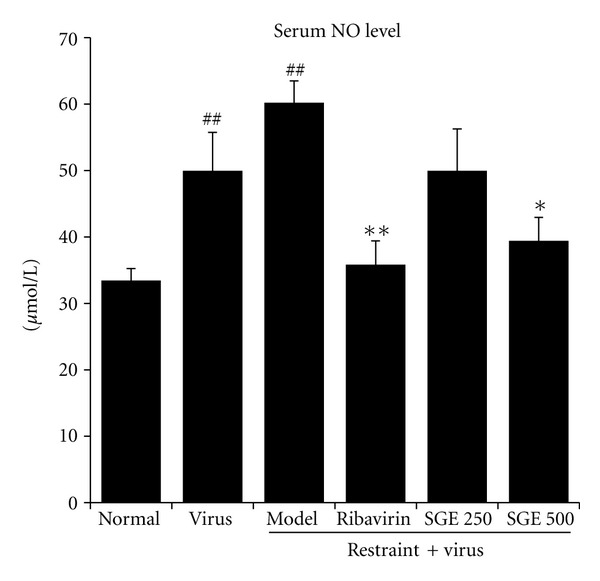
Effect of SGE on serum NO level in restraint-stressed mice after virus infection. Level of serum NO was determined by Griess test. The results represented the mean ± SE of values obtained from eight Kunming mice in each group. The significance of differences was from the normal control group at ^##^ *P* < 0.01 and from the model group at **P < 0.01, *P < 0.05.

**Figure 6:**
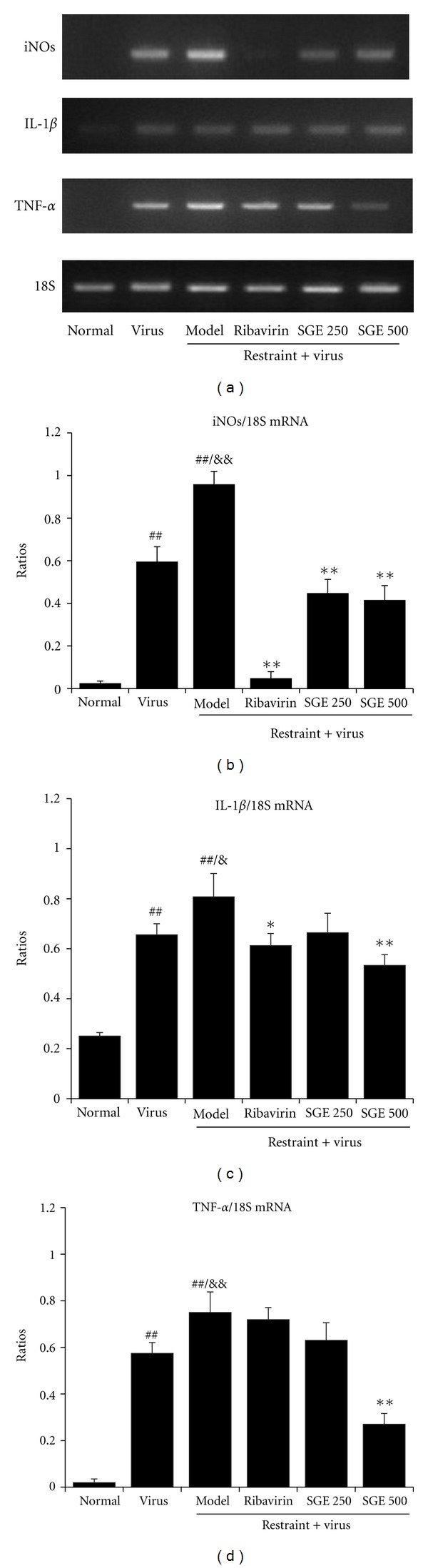
Effect of SGE on mRNA expressions of proinflammatory cytokines of lung tissues in restraint-stressed mice postinfection. iNOs, TNF-*α*, and IL-1*β* mRNA expression in lung tissues from 8 mice in each group were determined by RT-PCR and normalized by 18S. The significance of differences were from the normal control group at ^##^ *P* < 0.01, from virus control group at ^&&^ *P* < 0.01, ^&^ *P* < 0.05, and from the model group at *P < 0.01, *P < 0.05.

**Figure 7:**
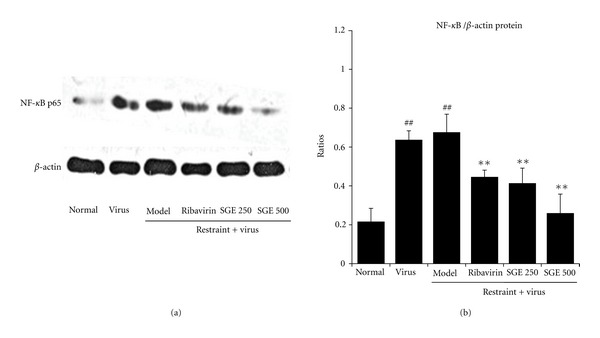
Effect of SGE on expression of nuclear NF-*κ*B p65 protein in restraint-stressed mice postinfection. Nuclear NF-*κ*B p65 protein levels in lung tissues from 4 mice in each group were determined by western blotting and normalized by *β*-actin. The significance of differences was from the normal control group at (^##^) *P* < 0.01 and from the model group at (**) *P* < 0.01.

To explore the effects of SGE on inflammatory reaction in influenza-induced pneumonia, we detected the concentration of nitrogen containing radical NO in serum and the mRNA levels of iNOs in lung tissues. At the same time, inflammatory markers such as IL-1*β* and TNF-*α* mRNA levels in lung tissues were also assayed. As shown in Figure 5, virus infection induced an increase of serum NO level. It was even higher in virus-infected restrained mice. However, oral administration of SGE (500 mg/kg/day) to infected mice could significantly decrease serum NO level when compared with model control (*P* < 0.05). Alteration in iNOs mRNA level was compatible with the above finding. In addition, as shown in Figure 6, the transcription levels of IL-1*β* and TNF-*α* in model controls were significantly increased, while SGE groups (500 mg/kg/day) obviously lowered both mRNA levels. However, IL-1*β* mRNA level was only slightly decreased in those treated with ribavirin and SGE (250 mg/kg/day). It is well known that the production of inflammatory markers was regulated by a transcription factor NF-*κ*B. We further examined the effects of SGE on NF-*κ*B activation. As shown in Figure 7, SGE could prevent nuclear accumulation of p65 (the major component of NF-*κ*B) in lung tissue of infected stressed-mice in a dose-dependent manner. Taken together, results demonstrated that SGE had protective effects against virus-caused pneumonia.

## 4. Discussion 

 In ancient China, traditional Chinese medicine (TCM) has played a key role in fighting the influenza pandemic [7]. TCM was performing well in clinical practice and showed potentials in the therapy of influenza [15, 16]. Although many TCM had been reported to have a valid anti-influenza effect [14, 17], there was limited data supporting this. There is an urgent need to find a suitable animal model for evaluation of anti-influenza activities of TCM. It is well known that antivirus drug and TCM work in a very different way upon the human body. Anti-virus drug target on virus and is ineffective in virus-induced complication. In contrast, TCMs are thought to work in a synergic way that interacts with the virus and the body based on the symptoms and keeps the body in a fine balance. Thus, in screening and evaluation of anti-influenza activities of TCM, host factors cannot be ignored. We also found that the effects of SGE against influenza were difficult to evaluate through general mouse model (Supplementary Figure 1 available online at doi:10.115/2012/236539). Some studies revealed that the pathogenesis of influenza virus infection in humans depends on a combination of virus and host factors [18]. The results in the present study showed that intranasal administration of influenza virus to normal mice could only partially cause sickness in the animals, not even to start the outbreak of pneumonia. Although there were individual differences in experimental animals, when the infected mice were loaded with restraint stress, the induction of sickness was found in all animals. Instead, the induction rate was only 80% for those without restraint stress. Sickness symptoms, such as shortness of breath, piloerection, rapid losing of body weight and becoming moribund, were being aggravated at 5 days after infection. Moreover, the mortality of animals was significantly increased from 30% to 78% with restraint stress. When we observed from the ventral view of intact lungs, all lobes displayed severe edema and hemorrhagic pleural effusion. Lung pathological slides showed fulminant viral pneumonia with substantial damage and severe inflammation in restraint stress-loaded infected mice. When we calculated the virus load in restraint stress-loaded infected mice, we found that virus persisted and could not be cleared quickly. The content of replicating virus in the lungs was nearly twofold higher than that of the normal mice when measured at 5 days post-infection. Therefore, enhanced pathogenesis of influenza was attributable to restraint stress and consequential changes in immune defense of the lungs. Heightened cytokine and chemokine levels are also hallmarks of severe influenza infection, which have been observed in both human and animal models [18, 19]. Recent studies showed that the levels of inflammatory cytokines and chemokines in lung or serum were correlated closely with the viral load [20]. When compared with normal mice, a remarkable, exaggerated proinflammatory response was discovered in the lung and serum of restraint stressed mice. This could be an indicative sign describing the extent of viral spread within the lungs. In previous study, restraint stress had significantly altered the balance of CD4^+^ T/CD8^+^ T cells and reduced NK cell activity [11]. Thus, when the animal was exposed to an unencountered influenza virus, the immune defense of the host was not able to act effectively. Indeed, restraint stressed mice might be more susceptible to viral infection by affecting related susceptibility genes expression levels, which could eliminate individual differences of animals. This animal model could clearly imitate the actual pathological condition of viral pneumonia that can be intuitively observed and detected. Therefore, our animal model provided an ideal condition for the evaluation of the effects of TCM on anti-influenza and its subsequent pneumonia.

In the present SGE study, we used restraint-stressed mice model to evaluate its effects against influenza and results have shown that oral administration of SGE to infected stressed-mice could reduce morbidity and facilitate the recovery from influenza in survived mice. During the administration of SGE, we found that treated mice were in a slightly better physiological condition and the survival rate was improved. Simultaneously, the survival time of mice was also significantly prolonged. From the view of intact lungs, SGE treatment could alleviate edema, red blood cell extravasation and hemorrhagic pleural effusion. We further found from lung pathological slides that SGE inhibited influenza virus-stimulated pulmonary morphological changes, including decreased structural damages in blood vessel and alveoli. Besides, data also showed that SGE could decrease virus load in lung 5 days after infection in restraint-stressed mice. These results suggested that the effect of SGE on reducing inflammation progression in lung might be related to the decreased virus load. However, direct anti-virus effect of SGE was not found in vitro (data not shown). 

Influenza virus infection in host could be divided into five stages, which are adsorption, invasion, replication, maturation, and release. When host cells were infected by influenza virus, the collectins in the respiratory tract and lung, surfactant proteins A and D (SP-A, SP-D) will recognize, bind, and neutralize the virus to prevent attachment of virus to the host cell [21, 22]. Some studies indicated that SP-A and SP-D deficiency would aggravate influenza virus infection [23–25]. Previous studies have shown that transcription factor NFAT (nuclear factor of activated T cells) regulates both SP-A and SP-D gene transcription, and NFAT in turn is negatively regulated by Akt [26, 27]. Stress could result in an increased expression of activated Akt, thus Akt activation may result in increased inhibition of NFAT and in turn lowered transcriptional activation of SP-A and SP-D [26, 27]. Apart from SP-A and SP-D deficiency, the increased ICAM-1 protein levels were also reported to aggravate virus infection [28]. ICAM-1 is an adhesion molecule found on the vascular endothelium and airway epithelial cells. ICAM-1 participates in leukocyte recruitment and it could be an entry site for certain viruses [29]. Some studies had showed that stress could induce the production of reactive oxygen species (ROS), while ROS was indicated to increase ICAM-1 levels [30]. In this study, we found that restraint stress obviously increased ICAM-1 mRNA expression and decreased SP-A, SP-D mRNA expression. This may be a potential mechanism for how restraint stress predisposes the respiratory tract to influenza infections. However, oral administration of SGE could restore and further strengthen SP-A, SP-D expression levels, as well as reduce the expression of ICAM-1, which were in favor of clearing influenza virus. When the escaped virus successfully infected host cell, the single-stranded RNA of the influenza virus is recognized by toll-like receptor 7 (TLR7) and retinoic acid-inducible gene-I (RIG-I). The pathway induces the production of type I IFNs and activate interferon-inducible transmembrane (IFITM) protein family, which further activates the immune system to help elimination of virus [18, 31]. The latest study found that IFITM3 prevented the morbidity and mortality associated with influenza [32]. Our results showed that restraint stress decreased IFITM3 mRNA expression, thereby impeding the transduction of cellular immunity signals. This delayed immune response after restraint stress is compatible with the previous findings, which showed that restraint stress could result in immunosuppression [11]. Recent evidence has also shown that SGE possess immunomodulation effects. However, oral administration of SGE could strengthen IFITM3 mRNA expression. Combined with the evidence that SGE could protect against immunocompromise in restraint-stressed mice [11], it can be inferred that SGE could improve immune function to help the host effectively clear influenza virus by increasing IFITM3 mRNA expression in restraint-stressed mice. 

In fact, influenza virus alone does not make critical contribution to mortality induced by influenza, but the “cytokine storm” produced by the disorder of immune response triggered by the virus can result in inflammatory reaction of lung tissues and fatal lung tissue injury. Our results showed that SGE could decrease the pro-inflammatory cytokine TNF-*α* and IL-1*β* mRNA levels in lung 5 days after infection. We further found that SGE prevented nuclear accumulation of NF-*κ*B p65 in the lungs. This finding is predictable as rosmarinic acid, caffeoylquinic acid, and caffeic acid, which are major active constituents found in SGE, have antiviral and anti-inflammatory effects [33–36]. Moreover, isofraxidin, another active constituent of SGE, has potent anticancer, antifatigue, antistress, immune-accommodating and cholagogic effects [37, 38]. Thus, the immunoregulating effects of SGE could help keeping a fine balance of the host immune system, which may be critical for viral clearance and inflammatory process relief. Several studies had considered the ability of SGE in improving immunologic response was attributable to its antioxidative capacity [11]. In addition, antioxidants were found to inhibit influenza A virus replication and influenza A virus-induced pro-inflammatory gene expression [39]. Our previous study suggested that SGE interferes with the oxidative stress [11]. In this study, we found that virus triggered iNOs upregulation and subsequent NO production was inhibited significantly by SGE treatment. Besides, the antioxidative property of rosmarinic acid has been demonstrated by its ability to reduce liver injury induced by D-galactosamine [40] and lipopolysaccharides [41] through the scavenging of superoxide molecules and the inhibition of cyclooxygenase-2 (COX-2) [42, 43]. However, severe influenza virus infections are associated with increased LTB4 [44]. The production of LTB4 was controlled by COX-2 [45]. Our previous studies demonstrated that SGE could reduce the release of LTB4 from neutrophils [46]. Our findings of lowered pro-inflammatory cytokines (IL-1*β*, TNF-*α*, and NF-*κ*B) in the lungs of infected-mice treated by SGE are compatible with the above. SGE might act as a selective COX-2 inhibitor in influenza virus infection.

Our study confirmed that SGE not only effectively decreased susceptibility of influenza in restraint-stressed mice, but also lowered virus-induced pro-inflammatory cytokines levels and improved pneumonia. These results might do contributions to the antivirus study of herbal medicines.

## Supplementary Material

The *Sarcandra glabra* extract (SGE) was performing well in clinical practice and showed potentials in the therapy of in*ﬂ*uenza. However, relative experiment data for this effective clinical consequence was limited through general animal model. In this study, we also evaluated the effects of SGE against H1N1 in*ﬂ*uenza in infected normal mice. Although, SGE treatment could improve health status when compared to virus control group. However, our study results showed that SGE couldn't reduce morbidity, mortality and prolong survival time. Thus, these data suggested that the effects of SGE against influenza were difficult to evaluate through general mouse model.

## Figures and Tables

**Table 1:** Sequences of primers used in RT-PCR.

Gene name	Sequence	Product size (bp)
NP	(F) 5′-CAGGTACTGGGCCATAAGGAC-3′	330
	(R) 5′-GCATTGTCTCCGAAGAAATAAG-3′	
SP-A	(F) 5′-CCTCTTCTTGACTGTTGTTG-3′	509
	(R) 5′-TTGTAATGCTTGCGATGG-3′	
SP-D	(F) 5′-GCCGAAGTGTTGGAGAC-3′	147
	(R) 5′-CTGTGATGAGTTGCTGTATG-3′	
ICAM-1	(F) 5′-CAGACGGAAGGCAGATG-3′	197
	(R) 5′-CCACAATGACCAGCAGTA-3′	
IFITM 3	(F) 5′-AAGCCTTCATCACCG-3′	169
	(R) 5′-AGGGACCAGACCACAT-3′	
IL-1*β*	(F) 5′-GCTGGAGAGTGTGGAT-3′	137
	(R) 5′-CTTGTGAGGTGCTGATG-3′	
TNF-*α*	(F) 5′-GGCGGTGCCTATGTCTC-3′	362
	(R) 5′-GCAGCCTTGTCCCTTGA-3′	
iNOs	(F) 5′-CCCAAGGTCTACGTTCAGGACA-3′	246
	(R) 5′-GGAAAAGACTGCACCGAAGATATCT-3′	
18S	(F) 5′-GGGAGAGCGGGTAAGAGA-3′	241
	(R) 5′-ACAGGACTAGGCGGAACA-3′	

F: forward primer; R: reverse primer.

**Table 2:** Effects of SGE on the morbidity rate of restraint-stressed mice postinfection.

Grouping	Morbid mice/total mice	Morbidity (%)	Mean times to sickness (day)
Normal control	0/10	0	>21.0
Virus control	8/10	80	10.5 ± 1.8^##^
Model control	9/9	100	7.2 ± 0.2^##/&&^
Restraint + virus			
50 mg/kg ribavirin	4/10	40	15.7 ± 2.4**
250 mg/kg SGE	9/11	82	9.7 ± 1.7
500 mg/kg SGE	9/11	82	10.0 ± 1.6*

Morbidity is presented as the percent of animals that exhibit sickness symptoms to the total number of mice. Time course of morbidity of each mouse was recorded until the 21st day after viral infection. The significance of differences was from the normal control group at (^##^)*P* < 0.01, from virus control group at (^&&^)*P* < 0.01, and from the model group at (**)*P* < 0.01, (*)*P* < 0.05.
