# Supplementary material for: Sarcandra glabra Extract Reduces the Susceptibility and Severity of Influenza in Restraint-Stressed Mice
Source: Evid Based Complement Alternat Med. 2012 Nov 1;2012:236539. doi: 10.1155/2012/236539 (PMC3511833; doi:10.1155/2012/236539)
Supplement: Supplementary file 1 — The Sarcandra glabra extract (SGE) was performing well in clinical practice and showed potentials in the therapy of inﬂuenza. However, relative experiment data for this effective clinical consequence was limited through general animal model. In this study, we also evaluated the effects of SGE against H1N1 inﬂuenza in infected normal mice. Although, SGE treatment could improve health status when compared to virus control group. However, our study results showed that SGE couldn't reduce morbidity, mortality and prolong survival time. Thus, these data suggested that the effects of SGE against influenza were difficult to evaluate through general mouse model. [file 236539.f1.doc]

**Supplementary information**

**Experimental design**

The experimental mice were randomly divided into five groups: normal control, virus control, positive control (virus + 50 mg/kg ribavirin), and two SGE groups (virus + 250 or 500 mg/kg SGE). Ribavirin and SGE were administered to mice by oral gavage for 10 consecutive days, while the rest of the groups received water only. On the 5th day of administration, mice were anesthetized by inhalation of ether vapor and then an approximate 2 × LD50 amount of virus (30 μl) was instilled into the nares. Experiment were conducted in duplicates of 10 mice for each group to observe daily changes in body weight, survival and several typical symptoms of illness, including rufﬂed fur, redness around the eyes, nose or mouth, hunched back, altered respiration and unresponsiveness were monitored for 15 days or until death.

**Results**

**Effects of SGE on influenza caused morbidity and mortality in mice.**

The effects of SGE against H1N1 inﬂuenza were evaluated in infected mice. After intranasal inoculation of influenza virus, mice were monitored daily for survival and weight changes. A lower survival rate was observed in virus control group as compared to normal control (*p* < 0.01, **Supplementary figure 1a**). Fifty percent of mice in the virus control group survived and the mean day to death (MDD) was decreased to 11.3±2.1 day when compared to normal control group (*p* < 0.01). Ribavirin (50 mg/kg) signiﬁcantly improved the survival rate to 90% and MDD to 14.3 ± 1.2 day (*p* < 0.05). However, we found that SGE could not improve the survival rate when compared to virus control group. Thereafter, the body weight of virus infected mice began to decrease on day 5 and dropped to a minimum on day 6 (**Supplementary figure 1b**). Survived virus infected mice started to gain weight on day 7, but virus control mice kept losing weight until day 8. SGE treatment at 250 and 500 mg/kg/day could signiﬁcantly improve health status of virus infected mice. Body weight changes were rather stable when compared to other groups, with slight weight lost at day 5. Furthermore, behavioral changes, such as a tendency to huddle, ruffed fur, altered respiration, and reduced food intake were observed in infected mice on the 5th day after virus infection. Morbidity was presented as percentage of morbid mice to total number of mice. As shown in **Supplementary table 1**, group differences in morbidity were evident over the 15-day post-infection period. Virus control experienced a 50% incidence in morbidity and mean time to sickness was 7.4 ± 1.6 day. SGE treatment at 250 and 500 mg/kg/day alleviated influenza symptoms and the incidence of morbidity was both 60%, while mean time to sickness was 7.2 ± 1.5 and 7.3 ± 1.7 day respectively. These results indicated that the anti-influenza effects of SGE can’t be evaluated correctly through normal mouse model.

**
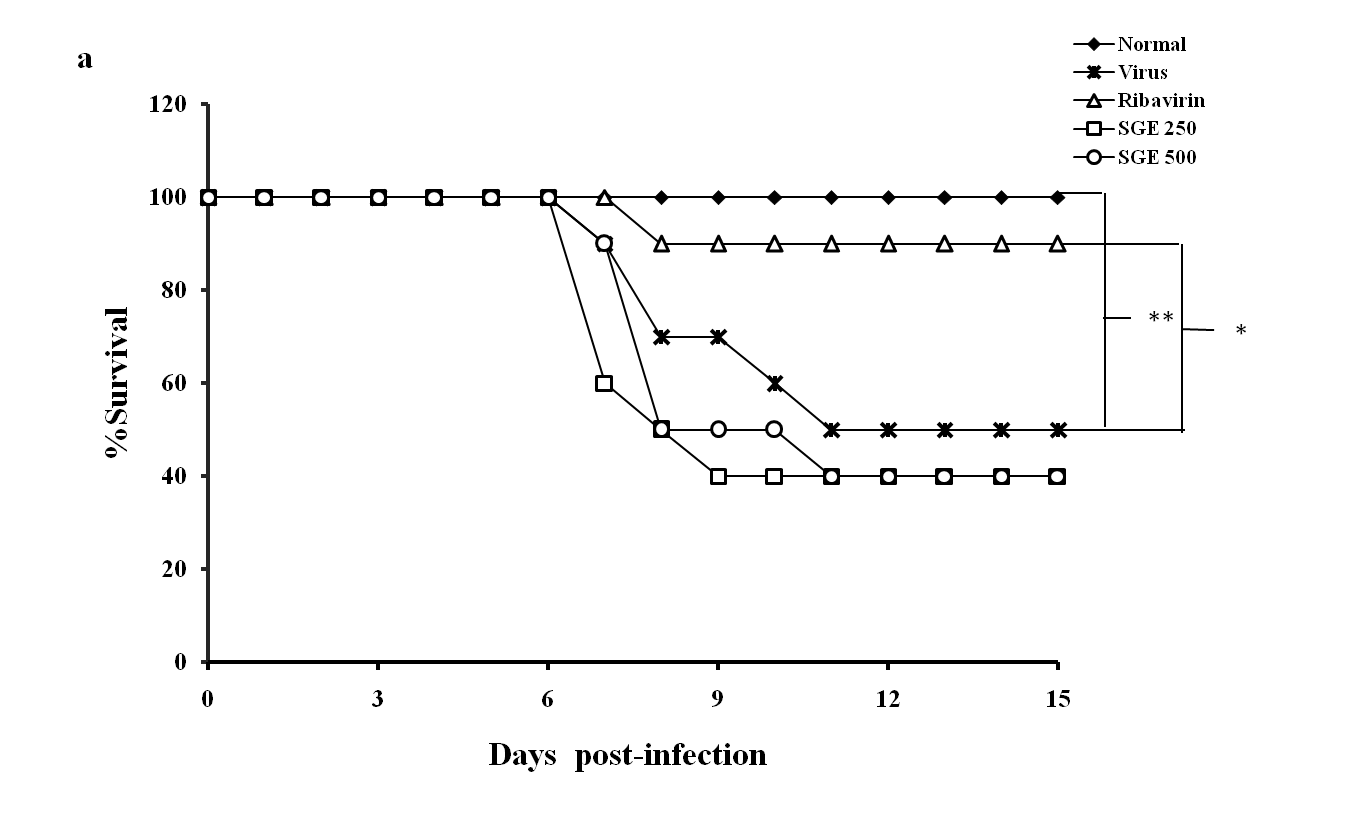
**

**
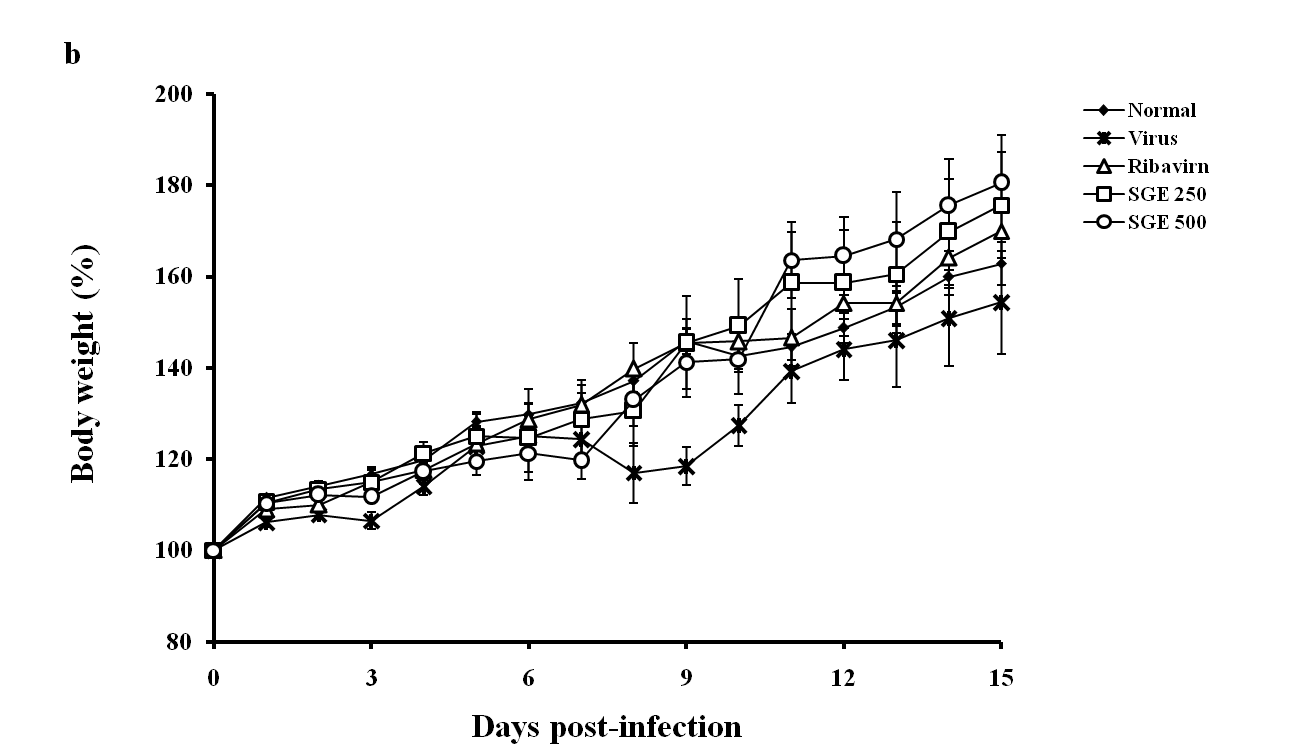
**

**Supplementary figure 1. Eﬀect of SGE on survival rates (a) and body weight change (b) of infected mice.** Mortality time course and survival days of each mouse were recorded until the 15th day after viral infection. Data were obtained from 10 animals in each group. (**) *p* < 0.01, (*) *p* < 0.05.

**Supplementary table 1. Effects of SGE on post infection morbidity rate of restraint-stressed mice.**

| **Grouping** | | **Morbid mice / total mice** | **Morbidity (%)** | **Mean times to sickness (day)** |
| --- | --- | --- | --- | --- |
| normal control |  | 0/10 | 0 | >15.0 |
| virus control |  | 5/10 | 50 | 7.4 ± 1.6^##^ |
| virus + | 50 mg/kg Ribavirin | 3/10 | 30 | 8.7 ± 2.2^&^ |
|  | 250 mg/kg SGE | 6/10 | 60 | 7.2 ± 1.5 |
|  | 500 mg/kg SGE | 6/10 | 60 | 7.3 ± 1.7 |

Morbidity is presented as the percentage of animals that exhibit sickness symptoms to the total number of mice. Morbidity time course for each mouse were recorded until the 15th day after viral infection. The signiﬁcance of diﬀerences was from the normal control group at (##) *p* < 0.01 and from virus control group at (&) *p* < 0.05.
